# 澳洲茄碱诱导肺癌细胞株H446凋亡及其机制探讨

**DOI:** 10.3779/j.issn.1009-3419.2015.07.05

**Published:** 2015-07-20

**Authors:** 文斯 黄, 颖 王, 海涛 朱, 荧荧 吴, 晓东 谢, 冬青 王

**Affiliations:** 212000 镇江，江苏大学附属医院 Affiliated Hospital of Jiangsu University, Zhenjiang 212000, China

**Keywords:** 澳洲茄碱, 凋亡, 肺癌细胞株H446, Solasonine, Apoptosis, Lung cancer cell line H446

## Abstract

**背景与目的:**

肺癌的发病率和病死率都急剧上升，小细胞肺癌首选化疗而不能手术，而中医药副作用小，有研究表明中药澳洲茄碱具有抗肿瘤活性作用。本研究旨在探讨澳洲茄碱对肺癌细胞株H446凋亡作用的影响。

**方法:**

用CCK8试剂盒筛选药物作用的合适浓度和时间，以药物浓度为0 μmol/L、3.4 μmol/L、6.8 μmol/L、13.6 μmol/L分4组，作用于H446细胞24 h后，倒置显微镜观察细胞形态的变化，DAPI核染观察细胞核变化，流式细胞术检测药物对细胞凋亡的影响，Western blot检测凋亡相关蛋白BCL2、BAX、CASP3表达的变化。

**结果:**

澳洲茄碱可降低H446细胞的存活率，抑制其增殖，具有剂量相关性，存活率可降低至16.77%(*P* < 0.001)，最高凋亡率为44.62%(*P* < 0.001)；H446有明显的细胞凋亡形态学变化；Western blot显示凋亡相关蛋白BAX、CASP3表达上调(*P* < 0.05)，凋亡抑制基因蛋白BCL2表达下调(*P* < 0.05)。

**结论:**

澳洲茄碱可抑制H446细胞的增殖，上调促凋亡相关蛋白表达，下调抑凋亡蛋白表达，从而促进细胞的凋亡。

## Abstract (en)

**Background and objective:**

Incidence and mortality rates of lung cancer are rising sharply. Small cell lung cancer patients prefer chemotherapy than surgery because of the insignificant side effects of Chinese medicine. Studies have shown that solasonine possesses an anti-tumor property. The aim of this study is to investigate the effect of solasonine on the apoptosis of lung cancer cell line H446.

**Methods:**

Appropriate concentration and time were selected with a CCK8 kit. The drug that was used in H446 cells was divided into four different concentrations: 0 μmol/L, 3.4 μmol/L, 6.8 μmol/L and 13.6 μmol/L. The changes in forms and nucleus of H446 cells that were stained with DAPI were observed under an inverted optical microscope. The effects on H446 cell apoptosis were detected by FCM. The changes in apoptosis-related proteins BCL2, BAX, and CASP3 were investigated using Western blot for 24 h.

**Results:**

Solasonine reduced the survival ratio of H446 cells and inhibited the proliferation with a dose-related effect. The survival ratio of H446 cells could be reduced to 16.77% (*P* < 0.001), and the highest apoptosis ratio was 44.62% (*P* < 0.001). Apoptosis was observed in H446 cells. Moreover, Western blot showed that the apoptosis-related proteins BAX and CASP3 were upregulated (*P* < 0.05).

**Conclusion:**

The proliferation of H446 cells can be inhibited by solasonine, and the expression of pro-apoptotic proteins is up-regulated, and the expression of anti-apoptotic proteins is down-regulated, thereby promoting the apoptosis of cells.

原发性支气管肺癌分小细胞肺癌(small cell lung cancer, SCLC)和非小细胞肺癌(non-small cell lung cancer, NSCLC)，其中SCLC占20%左右。在肿瘤中肺癌的发病率和病死率最高，且其发病率在全球范围内居高不下^[1]^。肿瘤的发生发展不仅与细胞增殖失控有关，还与细胞凋亡受阻相关，即增殖凋亡失衡的结果，其决定了肿瘤的生长速率，放疗和化疗可引起细胞坏死，一般的激素制剂、抗癌药物等是通过诱导肿瘤细胞凋亡的作用机制，从而达到治疗的目的。可见凋亡不仅在正常机体的正常发育、分化及死亡中产生重要影响，而且在肿瘤治疗方面也发挥重要作用，深受重视^[2, 3]^。多种中草药中，有些药已证明对肿瘤细胞有抑制作用。我国是个天然的药物大国，中草药的应用历史悠久，在治疗的药物中，抗肿瘤中成药的不良反应较小，因此抗肿瘤中成药成为了人们关注的治疗方法^[4]^。澳洲茄碱提取于中药龙葵，龙葵还包括龙葵碱(solanine)、澳洲茄边碱(solamargine, SM)，其为茄科草本植物，富含多种甾体生物碱，而生物碱类化合物多能促进肿瘤细胞的凋亡，肿瘤细胞凋亡通路的受阻与肿瘤的发生发展紧密相关^[5, 6]^。澳洲茄碱作用于肺癌的报道尚少，本研究旨在探讨澳洲茄碱对肺癌H446细胞凋亡的作用。

## 1 材料与方法

### 1.1 主要试剂与仪器

澳洲茄碱(纯度 > 98%)为上海亿林生物科技有限公司产品，胎牛血清(fetal calf serum, FBS)购于GIBCO公司，CCK8试剂、Annexin V/PI凋亡试剂盒均购于南京诺维赞生物技术有限公司，β-actin、CASP3、BAX、BCL2及辣根过氧化物酶标记的羊抗兔、羊抗鼠二抗则购于武汉博士德生物工程有限公司，分子净化工作台(苏州生化净化设备厂)，CO_2_培养箱及酶标仪(Thermo公司)，显微镜(Olympus公司)，流式细胞仪(Becton Dickinson公司)。

### 1.2 细胞培养

人肺癌H446细胞以10%胎牛血清的DMEM培养基(含青、链霉素各100 U/mL)，于37 ℃和5%CO_2_的细胞培养箱中进行培养。细胞单层贴壁状态，取对数生长期的细胞进行实验。

### 1.3 CCK8试剂检测细胞活性

收集呈对数生长的H446细胞，制成浓度为2.5×10^4^/mL的单细胞悬液，接种于96孔板中，每孔细胞数为5×10^3^个。细胞培养24 h后，弃原培养液，实验组每孔加入200 μL澳洲茄碱，浓度分别为0 μmol/L、3.4 μmol/L、6.8 μmol/L、13.6 μmol/L、27.2 μmol/L，只加培养基无细胞孔作空白对照组，每组设置5个复孔。将4块96孔板置于培养箱中分别培养3 h、6 h、12 h、24 h，弃原培养液，PBS洗一遍后，每孔中加入100 μL的CCK8与无血清培养基的混合液(比例1:10)，于37 ℃避光孵育1 h，在酶联免疫监测仪上以450 nm波长测定各孔吸光度(optical density, OD)值，记录并计算存活率及抑制率。

### 1.4 倒置显微镜观察细胞形态及DAPI核染后核变化

H446细胞培养于6孔板中，每孔1×10^5^个，0 μmol/L、3.4 μmol/L、6.8 μmol/L、13.6 μmol/L的澳洲茄碱作用24 h后，用PBS清洗两遍，4%多聚甲醛固定15 min，用0.1% triton-X 100处理5 min后每孔加入浓度为1 μg/mL的DAPI染液，常温避光孵育10 min，后用PBS清洗3遍后，于倒置显微镜观察细胞形态变化，荧光显微镜观察细胞核的变化。

### 1.5 流式细胞术检测细胞凋亡

将H446细胞常规培养于6孔板中，每孔1×10^6^，贴壁后与浓度为0 μmol/L、3.4 μmol/L、6.8 μmol/L、13.6μmol/L的澳洲茄碱共培养24 h。消化后离心5 min、1, 000 rpm，用预冷的PBS洗涤2遍，重悬细胞于100 μL的Annexin V结合缓冲液中，向每孔缓冲液中加入5 Μl Annexin V和5 Μl PI，将细胞放置室温下15 min，后向每孔中再加入400 μL的缓冲液，轻轻混匀，1 h内上机，记录并分析结果。

### 1.6 蛋白免疫印迹(Western blot)法检测凋亡相关蛋白的表达

以不同浓度(0 μmol/L、3.4 μmol/L、6.8 μmol/L、13.6 μmol/L)的澳洲茄碱处理H446细胞，24 h后收集，加入细胞裂解液(内含蛋白酶抑制剂和PMSF)，震荡离心，取上清测总蛋白量，100 ℃煮沸10 min后置于-20 ℃保存。每组取蛋白样品40 μg，于10%SDS-PAGE进行电泳，电泳后将凝胶的蛋白转移到PVDF膜上，将膜置于5%的脱脂奶粉中，常温下进行封闭处理1 h，孵育1:500稀释的抗体BCL2、BAX和CASP3，4 ℃过夜。膜经TBST(150 mmol/L NaCl, 10 mmol/L Tris, 0.1%Tween-20, pH7.6)洗涤3次，每次10 min，加入相应辣根过氧化物酶标记的二抗(1:2, 000)常温孵育1 h，再次经TBST洗涤3遍，每次5 min，用ECL化学发光液进行曝光^[7]^。

### 1.7 统计学分析

统计数据采用SPSS 16.0统计学软件进行分析，实验数据中计量资料用均数±标准差(Mean±SD)表示。多样本比较使用单因素方差分析，以*P* < 0.05为差异有统计学意义。

## 2 结果

### 2.1 澳洲茄碱抑制H446的增殖

通过CCK8实验的数据分析，浓度3.4 μmol/L、6.8 μmol/L、13.6 μmol/L、27.2 μmol/L加药组24 h细胞存活率分别为85.64%、64.68%、20.96%、16.77%，差异有统计学意义(*P* < 0.001)(图 1)，经过简单计数，对照组与浓度3.4 μmol/L、6.8 μmol/L、13.6 μmol/L、27.2 μmol/L加药组的细胞个数分别为5, 000个/孔、4, 326个/孔、3, 328个/孔、1, 248个/孔和998个/孔，与CCK8所得结果相近。

**1:**
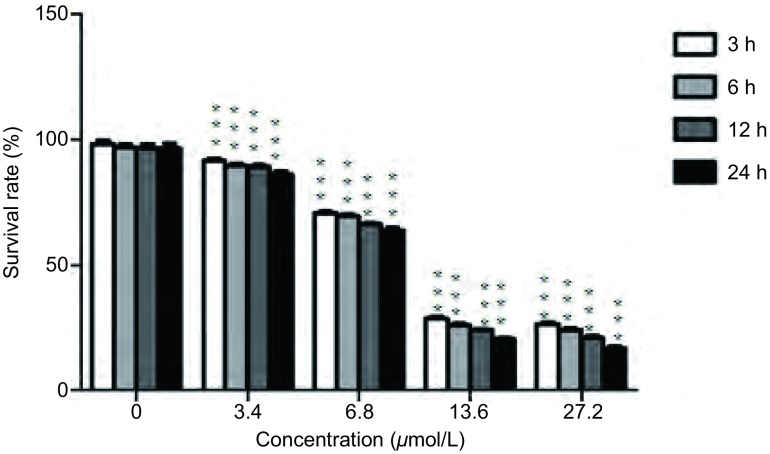
CCK8检测澳洲茄碱对H446细胞的增殖作用。分别用浓度为0 μmol/L、3.4 μmol/L、6.8 μmol/L、13.6 μmol/L、27.2 μmol/L的澳洲茄碱作用于H446细胞，于3 h、6 h、12 h、24 h用CCK8试剂检测。单因素方差分析，3 h、6 h、12 h、24 h的不同浓度与对应的对照组比较均有统计学意义，^***^：*P*≤0.001。 Detect proliferation of H446 by CCK8. With concentration of 0 μmol/L, 3.4 μmol/L, 6.8 μmol/L, 13.6 μmol/L, 27.2 μmol/L of solanine, CCK8 detected the proliferation of H446 cells in 3 h, 6 h, 12 h and 24 h. It had statistical significance compared with the corresponding controls in 3 h, 6 h, 12 h and 24 h analyzed with one-way analysis of variance, ^***^: *P*≤0.001.

### 2.2 澳洲茄碱诱导H446细胞凋亡

倒置显微镜观察，浓度为0 μmol/L、3.4 μmol/L、6.8 μmol/L、13.6 μmol/L的澳洲茄碱作用于H446细胞24 h后，对照组细胞形态正常，细胞核完整，而加药组细胞体积变小，核皱缩，轮廓不规整。DAPI染核后通过荧光显微镜观察，药物浓度越大，核变化越大，以13.6 μmol/L组变化最明显，细胞核固缩，边集(图 2)。流式细胞分析仪检测的结果，0 μmol/L、3.4 μmol/L、6.8 μmol/L、13.6 μmol/L的澳洲茄碱作用24 h后可出现细胞凋亡现象，流式细胞术检测的凋亡率与正常对照组相比明显增高，以浓度为13.6 μmol/L的作用尤为明显(*P* < 0.001)(图 3)。

**2:**
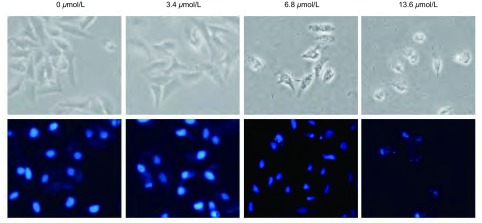
澳洲茄碱作用于H446细胞后细胞形态学变化(×100)。浓度为0 μmol/L、3.4 μmol/L、6.8 μmol/L、13.6 μmol/L的澳洲茄碱作用于H446细胞24 h后对细胞形态的观察及DAPI染色后观察细胞核的变化。 Change of cell morphology in H446 cells by solanine (×100). Incubated with 0 μmol/L, 3.4 μmol/L, 6.8 μmol/L, 13.6 μmol/L solasonine for 24 h, then detected the changes of H446 cells' morphology and nucleus staining by DAPI.

**3:**
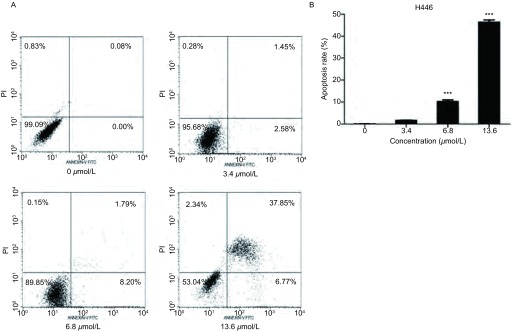
流式细胞术检测澳洲茄碱作用于H446细胞后的凋亡率。A：浓度为0 μmol/L、3.4 μmol/L、6.8 μmol/L、13.6 μmol/L的澳洲茄碱作用于H446细胞24 h后流式细胞术检测细胞凋亡率；B：细胞凋亡结果单因素方差分析，结果具有统计学意义。^***^：*P* < 0.001。 Detection of apoptosis rate of H446 cells incubated with solanine by flow cytometry. A: H446 cells was incubated with 0 μmol/L, 3.4 μmol/L, 6.8 μmol/L, 13.6 μmol/L of solanine for 24 h, apoptosis rate was detected by flow cytometry; B: The results analyzed by One-way analysis of variance showed that it had statistical significance. ^***^: *P* < 0.001.

### 2.3 澳洲茄碱影响凋亡相关蛋白的表达

通过Western blot法可观察到，随着药物浓度的增加，凋亡相关蛋白BAX、CASP3表达量增加，而凋亡抑制基因蛋白BCL2表达量减少，均以浓度为13.6 μmol/L的药物作用明显(*P* < 0.001)(图 4)。

**4:**
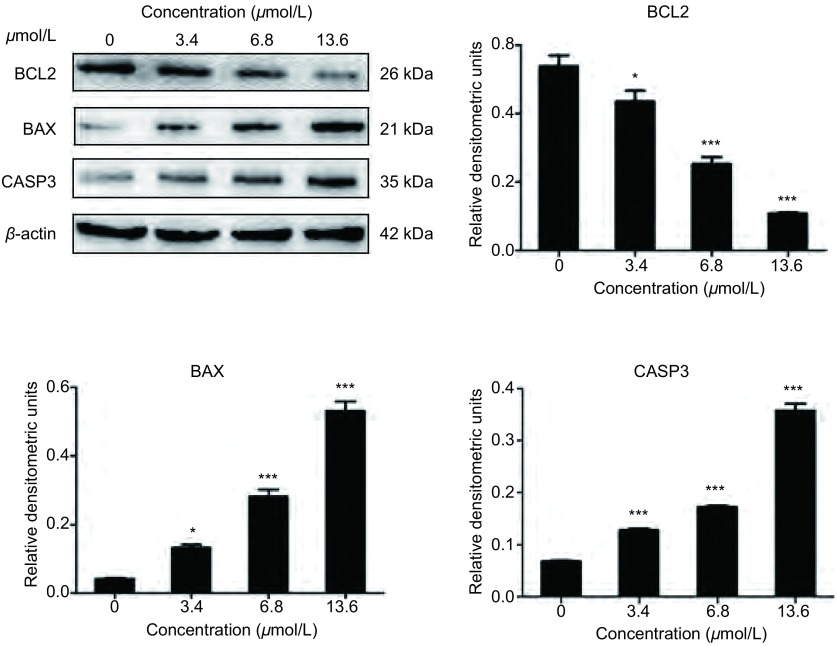
Western blot检测细胞凋亡相关蛋白的变化。A：用浓度为0 μmol/L、3.4 μmol/L、6.8 μmol/L、13.6 μmol/L的澳洲茄碱作用于H446细胞24 h后检测细胞内凋亡相关蛋白BCL2、BAX和CASP3表达量的变化；B、C和D：BCL2, BAX和CASP3蛋白Western blot灰度单因素方差分析表明，结果具有统计学意义。^*^：*P* < 0.05；^***^：*P* < 0.001。 Changes of apoptosis-related proteins detected by Western blot. A: After incubated with 0 μmol/L, 3.4 μmol/L, 6.8 μmol/L, 13.6 μmol/L of solanine, western blot was used to detect the changes of apoptosis-related proteins BCL-2, BAX and CASP3 in H446 cells; B, C and D: The one-way analysis of variance results showed that it had statistical significance. ^*^: *P* < 0.05; ^***^: *P* < 0.001.

## 3 讨论

澳洲茄碱提取于龙葵，有文献^[8, 9]^报道龙葵可抑制细胞突变，在肿瘤生长及增殖方面也有抑制作用，其可促进肿瘤细胞凋亡，并可抑制肿瘤转移，恢复细胞正常生理活动，增强机体免疫功能，调节机体免疫的同时具有细胞毒性作用。肺癌用中医解释，则为体内正气不足而邪毒外侵，所致气血瘀滞，内聚浊痰，故出现咳嗽咳痰、胸痛咯血等症状。龙葵恰好具有清热解毒、活血消肿的功效^[10]^，而澳洲茄碱作为龙葵的主要成分之一，也许可成为一个治疗肺癌的新方向。

肿瘤的发生和发展与肿瘤细胞的增殖凋亡失衡密切相关。在细胞的生理活动中，增殖与凋亡处于平衡状态，而凋亡是细胞死亡的主要调控程序^[11]^，人体中突变细胞的异常增殖、细胞凋亡的异常调节可促进肿瘤的发生。

本研究通过CCK8实验筛选出澳洲茄碱对H446细胞增殖抑制作用浓度及合适时间。实验得出澳洲茄碱对H446细胞的增殖抑制作用具有明显的浓度依赖性，而浓度为13.6 μmol/L与27.2 μmol/L作用相近，故在后续实验中选择0 μmol/L、3.4 μmol/L、6.8 μmol/L、13.6 μmol/L四个浓度。而实验筛选出的作用时间，不具有明显的时间依赖性，但以24 h作用抑制增殖效果最强，故选用24 h作为实验的最佳时间。上述实验结果表明，澳洲茄碱可降低H446细胞的存活率，减少其增殖，从而进一步对H446细胞凋亡产生作用。

澳洲茄碱对肺癌细胞H446凋亡的影响，通过对细胞形态学的观察、流式细胞术及凋亡相关蛋白(BCL2、BAX、CASP3)表达情况阐述其作用机制。

细胞形态学结果表明，澳洲茄碱作用于H446细胞后，细胞出现皱缩，细胞核固缩、边集、凋亡小体形成等细胞凋亡的表现，以药物浓度为13.6 μmol/L最明显，而对照组未见明显细胞形态变化；流式细胞术AnnexinV-FITC标记法对细胞凋亡比例的测定结果显示，澳洲茄碱可促进H446细胞的凋亡，并呈明显的浓度依赖，细胞早晚期凋亡率均升高。对凋亡相关蛋白(BCL2、BAX、CASP3)而言，BCL2家族是细胞凋亡信号转导的关键因素，其中BCL2是抑制凋亡基因的代表，而BAX是促进凋亡的基因，二者在肿瘤细胞凋亡程序中具有重要调控作用^[12, 13]^；CASP家族在细胞凋亡机制中占中心地位，其中CASP3是最重要的凋亡执行者，它活化后标志了细胞进入不可逆的凋亡阶段^[14, 15]^。本研究通过将澳洲茄碱作用于H446细胞后，应用Western blot实验得出抑制凋亡的基因代表BCL2表达下调，而CASP3表达上调，从而说明细胞处于凋亡不可逆状态，而BAX促凋亡基因编码的BAX蛋白增加进一步说明细胞走向凋亡。

综上所述，澳洲茄碱通过抑制H446细胞增殖，增加H446细胞的凋亡率，激活促凋亡基因，从而进一步上调促凋亡基因蛋白的表达并下调抑凋亡蛋白的表达，这可能是澳洲茄碱诱导肺癌细胞H446的凋亡机制。通过本研究及其他报道的实验结果，澳洲茄碱在临床肿瘤的治疗上可能有潜在的发展，值得进一步研究。
